# Quality of medical therapy in heart failure patients undergoing elective revascularisation: A protective effect of disease modifying therapy at discharge

**DOI:** 10.1038/s41598-017-15004-5

**Published:** 2017-11-02

**Authors:** Raphael Wurm, Martin Huelsmann, Marius Hienert, Veronika Seidl, Dominik Wiedemann, Guenther Laufer, Alfred Kocher, Christopher Adlbrecht, Martin Andreas

**Affiliations:** 1 Department of Surgery, Division of Cardiac Surgery, Medical University of Vienna, Vienna, Austria; 2 Department of Medicine II, Division of Cardiology, Medical University of Vienna, Vienna, Austria; 3 4th Department of Internal Medicine, Division of Cardiology, Hietzing Hospital, Teaching Hospital of the Medical University of Vienna, Vienna, Austria

## Abstract

The STICH(-ES) trial showed that coronary artery bypass grafting was superior to medical therapy alone in treating ischemic heart failure. However, dosages of disease modifying drugs were not reported. We included 128 (84% male, mean age 66 ± 11 years) consecutive patients with ischemic heart failure and an ejection fraction ≤35% undergoing isolated elective coronary artery bypass grafting. We defined optimal medical therapy (OMT) as prescription of ≥50% dosages of guideline recommended medications (i.e. beta-blocker (BB) and renin angiotensin system (RAS) antagonist) plus prescription of a mineralocorticoid receptor antagonist (MRA). The mean logistic EuroSCORE was 12.3 ± 13.8%. The five year survival was 74%. At discharge, 111 patients (87%) were on a BB and 106 (83%) were on a RAS antagonist. Forty-nine patients (38%) received an MRA. Only 8 patients (6%) received OMT. A Cox regression analysis revealed EuroSCORE (p < 0.001) and the use of MRA (p = 0.003) and BB (p = 0.037) at discharge as significant predictors of 5 year survival. Prescription rates of heart failure medication are comparable to those reported in the STICH trial, but rates of OMT are very low at admission and discharge. Prescription of BB and MRA was associated with improved survival, highlighting the need for disease management programs and rigorous discharge management.

## Introduction

The Surgical Treatment for Ischemic Heart Failure (STICH) trial enrolled patients with reduced left ventricular ejection fraction (LVEF) and coronary artery disease who were candidates for coronary artery bypass grafting (CABG)^1^. It compared a conservative strategy of medical therapy alone versus CABG and medical therapy, and found a benefit for CABG regarding relevant secondary endpoints such as death from cardiovascular cause and hospitalization for cardiovascular reasons and a statistically significant mortality benefit in the prespecified long-term analysis^1,2^. Patients in the STICH trial were treated with renin angiotensin system inhibitors and beta blockers in almost 90% of the cases, but dosages of heart failure specific drugs were not reported.

It has been shown that adherence to guideline-specific medical therapy is a strong predictor of outcome in chronic heart failure^3^. The results of STICH should be interpreted such that medical and surgical options are not competitors, but rather complementary strategies. Hence, not only the prescribed drugs but also their dosages could have important effects on outcome.

Guidelines recommend early discharge management in patients with acute heart failure with an emphasis on initiation and planning of therapy^4^. Data for patients with heart failure undergoing cardiac surgery are completely lacking. Here, we sought to evaluate the intensity, i.e. the percentage of recommended target dose, and the effect of medical therapy with heart failure specific drugs in a STICH-eligible population under “real world” conditions at a tertiary care university center.

## Methods

Consecutive patients suffering from ischemic heart failure with a LVEF of ≤35% who underwent elective CABG at a tertiary care center between 2009 and 2013 were included in this study. Exclusion criteria were acute surgery and concomitant valve replacement or reconstruction. Baseline evaluation was performed on the day of admission early in the morning. Patients had taken their morning medication, vital signs were taken and blood was drawn after patients had rested in a supine position.

Mortality status was assessed through the Austrian statistic agency (Statistics Austria) as well as by telephone follow-up and review of patient files in hospitals of the same city.

The analysis of heart failure specific medical therapy was based on a prospectively maintained database. Heart failure specific medication was grouped according to the dosage prescribed as percentage of the dosage recommended in the guidelines for the treatment of chronic heart failure by the European Society of Cardiology^4,5^. Group 1: not prescribed; group 2: 1–49% of recommended target dosage; group 3: 50–99% of recommended target dosage; group 4: 100% of recommended target dosage. For this study, optimal medical therapy (OMT) was defined as at group 3 or 4 for (1) beta blocker (BB) and for (2) renin angiotensin system (RAS) antagonists plus (3) any prescription of a mineralocorticoid receptor antagonist (MRA). This is based on the current guidelines and a prospective trial showing that while higher dosages are beneficial, there was no difference between 50–99% and 100% of target dosage^4,6^. It was measured by analyzing the prescribed dosages at admission and discharge. Continuous variables are presented as mean and standard deviation and compared by the independent samples *t*-test. Total numbers and proportions are reported for categorical outcomes and tested by the Chi-square test. Heart failure therapy prescription at admission and discharge was compared by the Wilcoxon signed-rank test. The effect of heart failure therapy prescription on survival was assessed with a Cox-regression analysis including therapy prescription at discharge from primary hospital stay (BB, RAS antagonist, MRA) and the logistic EuroSCORE. SPSS Statistics (IBM SPSS Statistics for Mac, Version 23.0. Armonk, NY: IBM Corp.) was used for statistical analysis. A two-sided p-value less than 0.05 was considered as significant.

The datasets generated during and/or analysed during the current study are available from the corresponding author on reasonable request.

## Results

The study population consisted of 128 consecutive patients. Baseline demographic data, clinical characteristics and risk factors are reported (Table 1). Dosages of RAS antagonists decreased significantly (p = 0.026), whereas the MRA prescription rate increased significantly (p < 0.001) during the hospitalization (Table 2). More than 80% received a BB and a RAS antagonist at discharge. Eight patients (6%) fulfilled the OMT criteria at admission, 14 (11%) at discharge (p = 0.21).

**Table 1:** Patient characteristics regarding demographic and risk factors.

Demographic/laboratory		Risk factors	
Age (years)	65 ± 11	COPD	19%
Gender (female/male)	16% / 84%	Peripheral artery disease	34%
Body mass index (kg/m^2^)	27.8 ± 4.2	Cerebrovascular disease	27%
Systolic BP (mmHg)	126 ± 21	Smoking (all time)	38%
Diastolic BP (mmHg)	72 ± 12	Diabetes	43%
Heart rate (bpm)	78 ± 20	Dyslipidemia	66%
Ejection fraction (%)	26 ± 5	Additive EuroSCORE	7 ± 3
Hemoglobin (mg/dl)	13.9 ± 11.7	Logistic EuroSCORE	12.3 ± 13.8
Creatinine (mg/dl)	1.5 ± 1.6		
NT-proBNP (pg/ml)	3464 ± 4855		

Caption: BP: blood pressure; NT-proBNP: n-terminal prohormone of brain natriuretic peptide; COPD: chronic obstructive pulmonary disease.

**Table 2:** Heart failure therapy and statins during the primary admission

Therapy	Timepoint		Dose/Prescription			
*% of recommended target therapy*			*0%*	*1–49%*	*50–99%*	*100%*
**Beta-blocker**		admission	18 (14%)	49 (38%)	43 (34%)	17 (13%)
p = 0.628		discharge	17 (13%)	49 (38%)	51 (40%)	10 (8%)
**RAS Antagonist**		admission	18 (14%)	37 (29%)	41 (32%)	31 (24%)
P = 0.026		discharge	22 (17%)	48 (38%)	33 (26%)	25 (20%)
***Daily prescription***			***No***		***Yes***	
**MRA**	admission		100 (78%)		28 (22%)	
p < 0.001	discharge		79 (62%)		49 (38%)	
**Statin**	admission		30 (23%)		97 (76%)	
p = 0.071	discharge		38 (30%)		90 (70%)	

Caption: RAS: renin angiotensin system; MRA: mineralocorticoid receptor antagonist.

**Figure 1:**
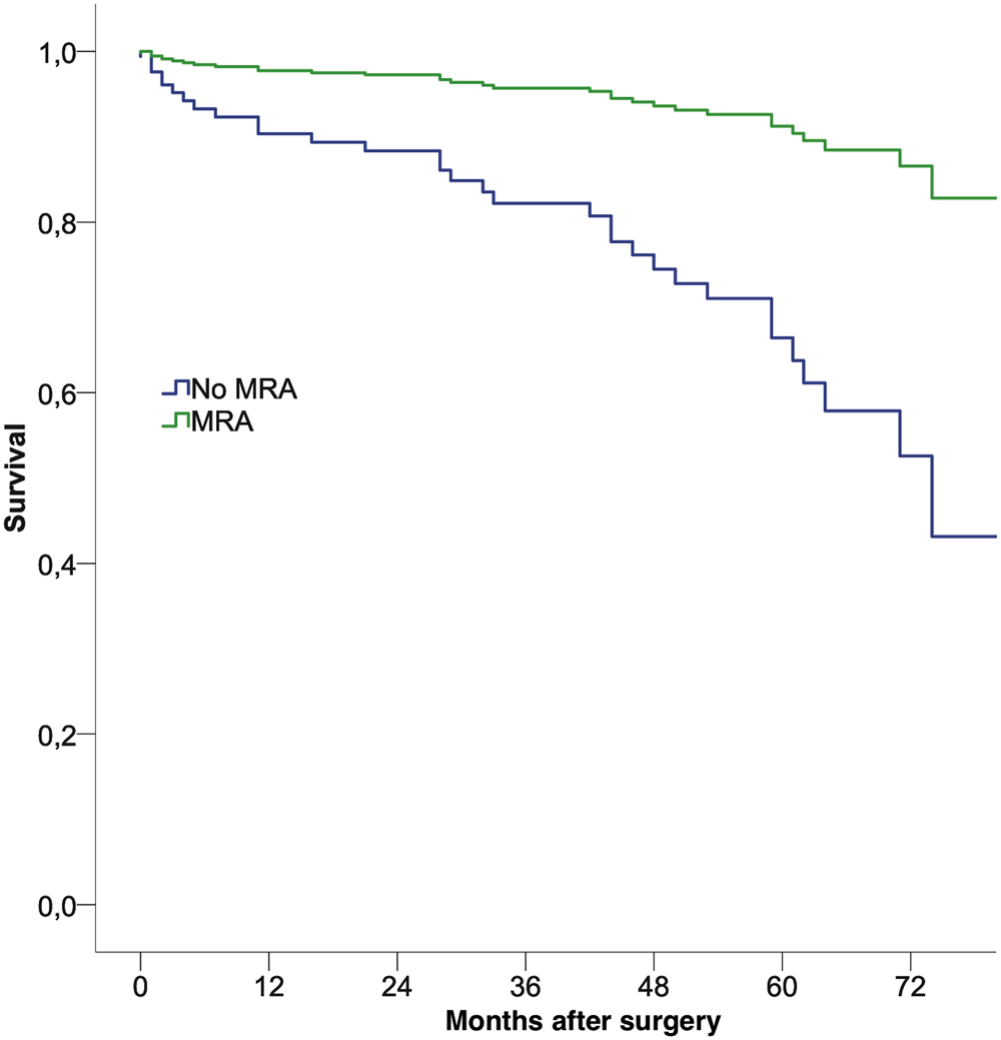
Cox regression analysis demonstarting the effect of an MRA at discharge on long-term survival (p = 0.003), corrected for preoperative risk.

**Figure 2:**
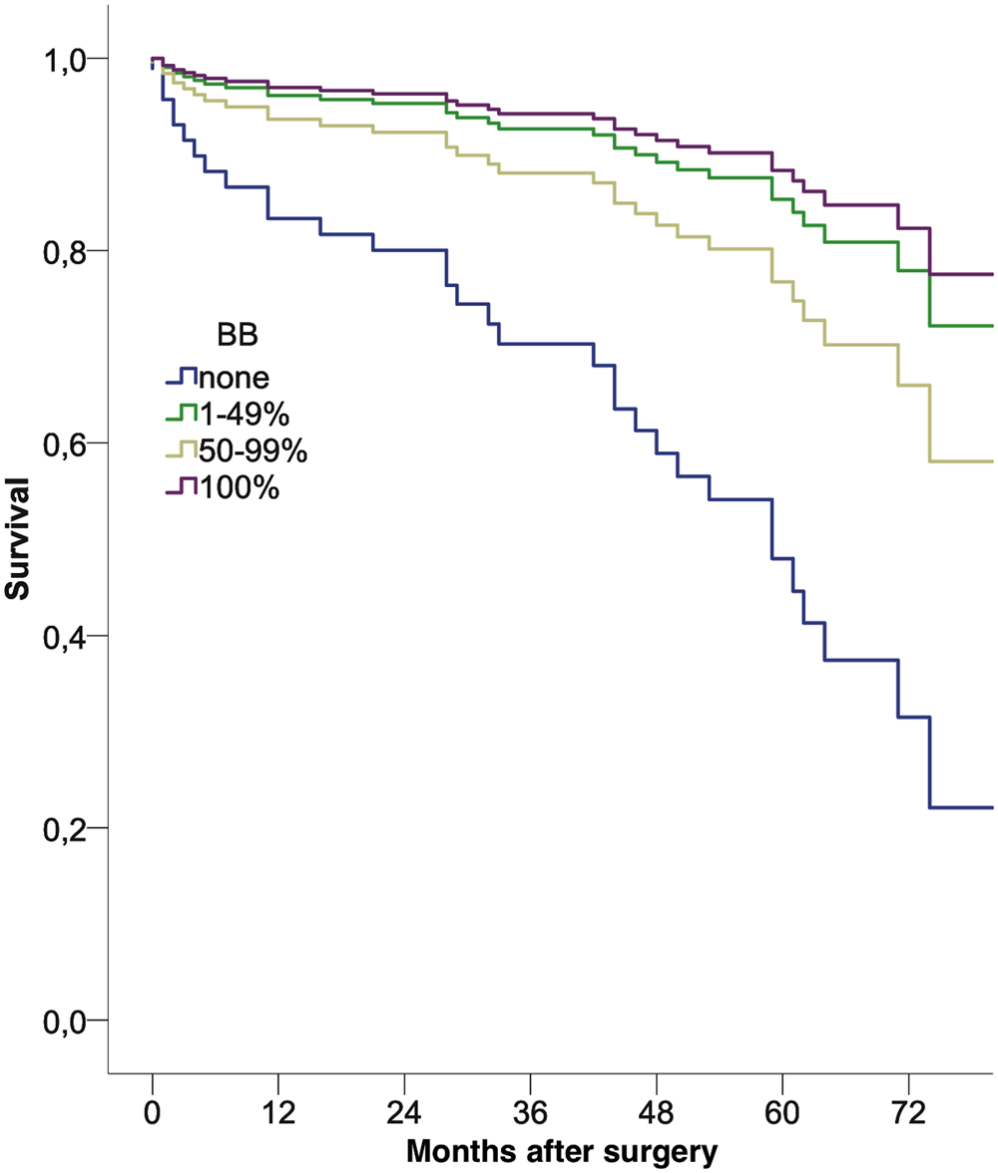
Cox regression analysis demonstarting the effect of beta-blocker dosage at discharge on long-term survival (p = 0.037), corrected for preoperative risk.

Of the 128 patients, two patients (1.6%) died within 30 days after surgery. One patient was lost to follow-up due to geographical reasons. After a mean follow-up period of 47 ± 22 months, 34 patients (27%) died. The one and five year survival was 91% and 74%, respectively. A Cox-regression analysis including medication at discharge and preoperative logistic EuroSCORE, revealed the use of MRA or BB as well as the preoperative EuroSCORE as independent predictive factors (Table 3, Figs 1, 2). Beta blocker therapy was predictive in patients receiving a low, high and full dose compared to no BB at discharge (Table 3, Fig. 2).

**Table 3:** Cox-regression analysis including medical therapy at discharge and logistic EuroSCORE.

Factor	beta	95% CI	p-value
EuroSCORE	1.053	1.033–1.074	<0.001
MRA	0.224	0.033–0.857	0.003
BB			0.037
*BB 1–49%*	*0*.*216*	*0*.*073–0*.*643*	*0*.*006*
*BB 50–99%*	*0*.*360*	*0*.*130–1*.*000*	*0*.*050*
*BB 100%*	*0*.*169*	*0*.*033–0*.*857*	*0*.*032*
ACEI/ARB			0.113
*ACEI/ARB 1–49%*	*0*.*421*	*0*.*134–1*.*323*	*0*.*139*
*ACEI/ARB 50–99%*	*0*.*366*	*0*.*108–1*.*236*	*0*.*106*
*ACEI/ARB 100%*	*1*.*149*	*0*.*341–3*.*865*	*0*.*823*

Caption: ACEI: angiotensin converting enzyme inhibitors; ARB: angiotensin receptor blockers; MRA: mineralocorticoid receptor antagonist.

## Discussion

The present study assessed the quality of perioperative heart failure specific medical therapy in a STICH-eligible population under “real world” conditions at a tertiary center. We found a very low OMT prescription of 6% at admittance that was only mildly attenuated during hospital stay to reach 11% at discharge. The positive effect of drug prescription at discharge on mortality highlights how important optimization of prescription rates and up-titration in this collective is.

Heart failure patients undergoing CABG for revascularization represent an important population at risk for death or hospitalization. This is underlined by an increase in heart failure related hospital admissions after CABG in the last 20 years, especially in patients with a LVEF ≤ 35%^7^. While STICH showed that CABG is superior when compared to medical therapy alone with regards to important secondary endpoints and in the primary endpoint all-cause mortality in prespecified long-term analysis, medical therapy has certainly consolidated its position as the cornerstone of heart failure therapy^8^.

The high-risk profile of the population included in the present study is evidenced by both the reduced LVEF and the increased NT-proBNP levels, which are representative of a population with severe heart failure^9–11^. However, a median systolic blood pressure of 126 and a median heart rate of 78 suggest that maximum tolerated dosages of disease-modifying drugs were on average not achieved.

In the light of this finding, one might argue that heart failure therapy should be uptitrated between decision to surgery and admission for surgery. However, the uptitration of heart-failure specific medication should be performed very carefully in patients with significant coronary artery disease prior to coronary artery bypass surgery. First, lower perfusion pressure may increase ischemia in myocardial tissue. Furthermore, RAS antagonists may lead to significant vasodilatation during cardiopulmonary bypass and were reported to increase operative mortality if not stopped 24 h prior to surgery^8^. It is possible that despite relatively high mean heart rate in our sample, some patients were on maximum tolerated dosages that were individually smaller than 50% of the guideline recommended dose. Also, immediate uptitration after surgery is sometimes purposefully delayed until after cardiac rehabilitation because the increase in exercise capacity would allow for higher dosages^12^.

The influence of drug therapy at admission on perioperative mortality could not be assessed due to a very low perioperative mortality rate. Survival was 85% after three years of follow-up, which is slightly higher than the 78% reported in the STICH trial, despite the STICH patients being 7 years younger on average^1^.

Prescription of a BB and/or MRAs at discharge had a significant protective effect on long-term survival, which was independent from preoperative risk. Furthermore, we observed a strong trend towards an improved outcome in patients who were prescribed higher dosages of BB (Fig. 2). Interestingly, we also found a trend towards an improved survival in the group receiving 1–49% of target dosage compared to that receiving 50–99%. A large trial investigating the effect of BB dosages found the group receiving the highest dosage (defined as 51% or more) to have the best prognosis^13^. As such, this result is likely to be due to the smaller sample size of the population studied here.

These observed therapeutic effects of BB (Fig. 2) and MRAs (Fig. 1) are in line with several clinical trials in heart-failure patients, which culminated in the current guidelines, but were never shown in a surgical cohort^12^. These are the first detailed data in patients in STICH-like patients after surgical coronary revascularization. Beta blocker therapy after cardiac surgery previously revealed a strong protective effect, although specific doses were not accounted for and this cohort was not confined to heart failure patients^14^. We were, however, not able to show an effect of postoperative prescription of RAS antagonists on long-term survival. This is in the line with Ouzounian *et al*.^15^, who found no association between preoperative RAS antagonist therapy and short- or long-term survival in CABG patients

Prescription of OMT at admission was extremely low and we observed a non-significant increase at discharge that was mostly driven by an increase in MRA prescription. The fact that more than 90% of advanced heart failure patients were not prescribed OMT at admission is evidence for structural problems in caring for these patients. This is in accordance with other data reporting an underuse of OMT in more than 30000 heart failure patients in Austria^16^. Systematic disease management programs as recommended in current guidelines could be one way to address this problem.

Renin angiotensin system antagonists and BB were rarely modified during the hospitalization in our population, foregoing a potential synergy between surgical and pharmacological improvements, as evidenced by the association of discharge BB dosage and outcome. Individually, the rates of BB, RAS antagonist and MRA prescription are similar to those achieved in STICH. However, the intersection of these three that defines OMT is very small in our population, and this was not reported in STICH.

## Limitations

Even though the population studied here is comparable in size to studies conducted in a similar setting, our results are limited by the sample size in addition to its single center setting and retrospective non-randomized design^14^. However, the findings are supported by similar results in the general heart failure population showing that adherence to guideline recommended therapy and adherence to guideline recommended dosages is associated with improved outcome^3,17^. Larger trials are warranted to corroborate our findings in surgical heart failure patients.

## Conclusion

Prescription rates of heart failure specific medication are comparable to those reported in the STICH trial in this smaller sample of a real world population. An overwhelming majority is not prescribed an OMT. Prescription of BB and MRA at discharge is associated with improved outcome after surgery. This highlights the need for disease management programs and for a rigorous discharge management in surgically treated heart failure patients.

## Ethical approval

All data collected in this study were in accordance with the ethical standards of the institutional and with the 1964 Helsinki declaration and its later amendments or comparable ethical standards. The ethics committee of the Medical University of Vienna approved the study (EK Nr. 1087/2011). The need to obtain informed consent was waived by the EC.
